# Selenofolate inhibits the proliferation of IGROV1 cancer cells independently from folate receptor alpha

**DOI:** 10.1016/j.heliyon.2021.e07254

**Published:** 2021-06-05

**Authors:** Ali Razaghi, Antje Maria Zickler, Julian Spallholz, Gilbert Kirsch, Mikael Björnstedt

**Affiliations:** a Division of Pathology, Department of Laboratory Medicine, Karolinska Institute, Karolinska University-Hospital, SE-14152, Stockholm, Sweden; b Université de Lorraine, CNRS, L2CM, F-57000, Metz, France

**Keywords:** Folate, Selenofolate, Folate receptor, Cancer, Selenotherapy

## Abstract

Cancer is one of the main causes of human mortality worldwide and novel chemotherapeutics are required due to the limitations of conventional cancer therapies. For example, using redox selenium compounds as novel chemotherapeutics seem to be very promising. The objective of this study was to explore if folate could be used as a carrier to deliver a newly synthesised selenium derivative selenofolate into cancer cells. Particularly, the cytotoxic effects of this selenofolate compound were investigated in a variety of cancer cell types including lung, liver, and cervical cancers and specifically IGROV1 cells. Our results showed that selenofolate inhibits the growth of cancer cells *in-vitro*. However, despite the expectations, folate receptor alpha (FRα) was not involved in the transportation of selenofolate compound into the cells *i.e.* growth inhibition was independent of FRα, suggesting that multiple transporters (*e.g.* reduced folate carrier-1) are possibly involved in the delivery and internalisation of folate in IGROV1 cells. Additionally, selenofolate did not exert cell death through apoptosis. Instead, anti-proliferative activity showed to be the main cause of growth inhibition of selenolofate in the IGROV1 cell line. In conclusion, selenofolate inhibits the growth of cancer cells and thus, may be explored further as a potential chemotherapeutic agent.

## 1 Introduction

Cancer is one of the main causes of human mortality with a world-wide economic toll of 1.4 trillion USD per year. The traditional cancer treatments demonstrate limitations due to their side-effects and recurrence of the disease. Therefore, novel cancer therapies and new anticancer agents are highly sought after [1]. Selenium compounds as chemotherapeutic agents are promising due to their redox capacity to generate superoxide, (O_2_^-^). *i.e.* cancer cells are vulnerable to reactive oxygen species (ROS) because cancer cells produce a higher level of ROS [2]. Furthermore, in malignant cells, selenium often induces apoptosis at concentrations that do not affect the viability of normal cells. Selenium can reduce the mortality of colorectal, lung and prostate cancer depending on the concentration and chemical form [3].

**Figure 1:**
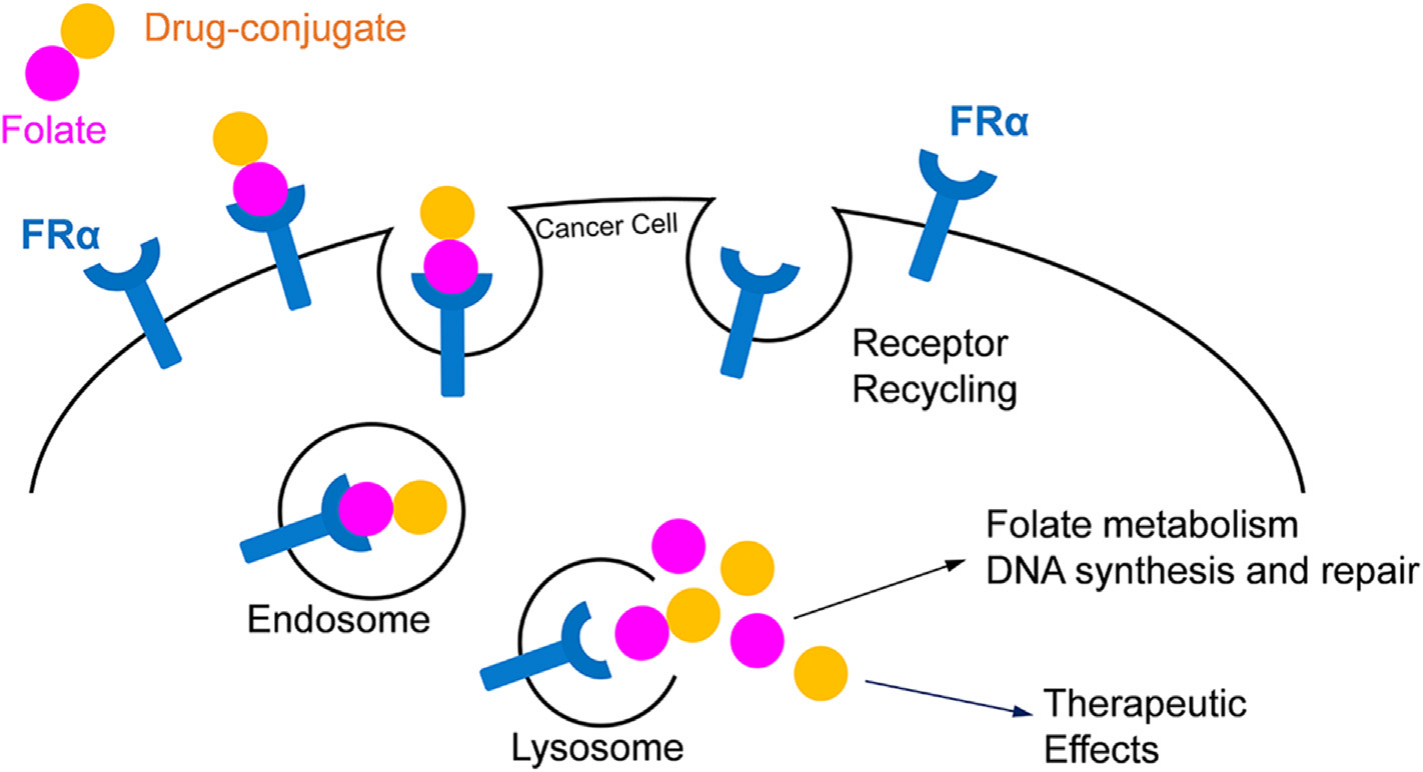
Schematic model of FRα used as a target in cancer therapy.

Folate (vitamin B9) and its oxidised form folic acid [4] take part in one-carbon transfer reactions which are essential for cell proliferation including DNA biosynthesis, cell division, growth, and survival. It has been shown that folate analogues, *e.g.* methotrexate, are significantly consumed by proliferating tumour cells [5]. Folate binds and is transported by a specific cell membrane-associated folate receptor (FR) [6]. The group of FRs consist of four isoforms (α, β, γ and δ). The FRα and FRβ isoforms are cysteine-rich glycolipid-anchored proteins while the FRγ is a soluble protein [6]. FRδ is only expressed on oocyte membranes and regulatory T-cells [7, 8]. FRα, the most explored FR, is encoded by the *FOLR1* gene. FRα is expressed at low levels on the apical surface of most normal cells. However, cancer cells, like endometrial, ovary, lung, cervix, colorectal, testicular choriocarcinoma mesotheliomas and renal cell carcinomas show over-expression of FRα [6]. In addition, accumulation of folate by cancer cells with a high level of FRα indicates that folate could participate in the progression of carcinomas [5]. Due to the high-level expression, FRα is an attractive therapeutic target for the development of novel anti-cancer agents in order to limit toxic side-effects on off-target tissues [5, 6]. Mechanistically, FRα captures extracellular folate and delivers it intracellularly by endocytosis. Thus, FR-mediated endocytosis might be used as a potential therapeutic pathway by conjugating the folate molecule to a chemotherapeutic agent [6, 9]. For instance, the variety of folate-conjugated drugs (*e.g.*, low molecular weight cytotoxic agents, anti-sense oligonucleotides, liposomes containing drugs and immunotherapeutic agents) have been successfully delivered to FRα-positive cancer cells [5]. After the entry of folate or a folate-conjugated drug into the cell, the FR protein is recycled back to the plasma membrane, permitting the start of a new cycle. At the same time, the drug-conjugate is released inside the cell to display cytotoxicity (Figure 1) [6]. For this reason, there has been growing interest in recent years in using folate as a carrier for drug delivery in cancer treatment. The use of folate conjugate drugs has been extensively reviewed elsewhere [10, 11].

In addition to FRs, entry of folate into the mammalian cells is also mediated by other transport processes namely reduced folate carrier-1 (RFC1) and proton-coupled folate transporter (PCFT). RFC1 (gene: *SLC19A1*) functions as a facilitative anion exchanger and transports anionic reduced folates, such as N5-methyltetrahydrofolate *via* a co-transport membrane system in which folate is exchanged for organic phosphates or sulphates [12]. PCFT (gene: *SLC46A1*) is another folate transport protein. Initially recognised as a heme carrier protein 1 (HCP1) similar to RFC1, PCFT is also a membrane transporter protein. PCFT mediates folate transport by the influx of one proton (H^+^) per transport cycle [12].

The primary objective of this study, therefore, was to explore if folate could be used as a carrier to specifically deliver redox selenium to cancer cells. In particular, the cytotoxic effects of a newly synthesised selenofolate compound as a potential chemotherapeutic agent was investigated in a variety of cancer types including lung, liver, and cervical cancer cell lines. Particularly, our study employed the IGROV1 cell line as a commonly used model for folate receptor targeting studies [13]. Our results showed that this novel selenofolate inhibits the growth of cancer cells *in-vitro*. However, despite our expectations, FRα was not involved in the transport and induction of cell death by this selenofolate compound. Showing promising anti-proliferative activity against cancer cell lines, it would seem, therefore, that further investigation is required to discover the mechanism of growth inhibition and also other potential transport pathways that can be employed to deliver this selenofolate chemotherapeutic to target tumour cells.

## 2 Material and methods

### 2.1 Synthesis of selenofolate and preparation of chemicals

**Figure 2:**
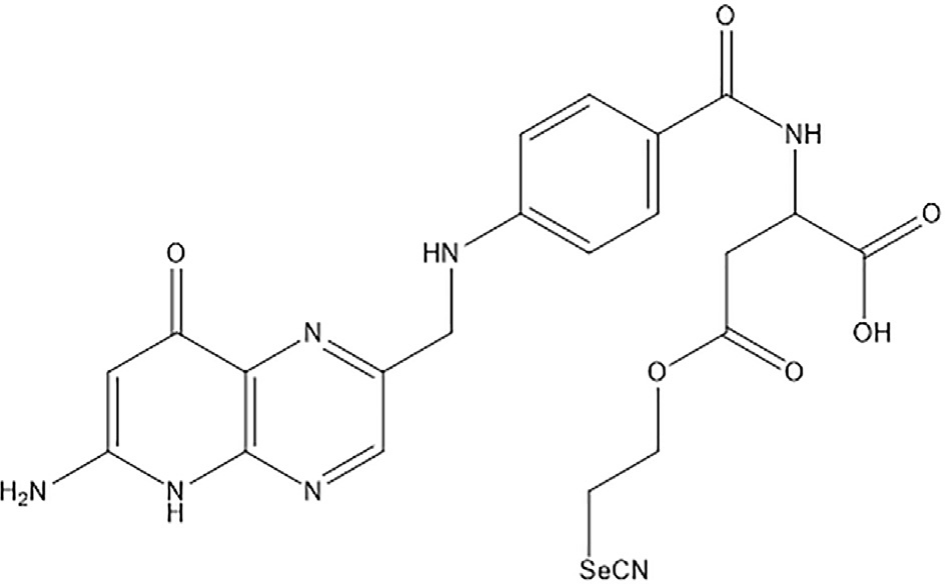
The formula of the chemical structure of the synthesised selenofolate compound [14].

Selenofolate was synthesised following the previously described procedure [14]. Folic acid (syn. folate) (Sigma, F7876), methotrexate hydrate (MTX) (Sigma, 133073-73-1) and selenofolate were solubilised in sterile ddH_2_O and pH-adjusted with 23% HCl and 1M NaOH to a final pH of 7.5 at a concentration of 10 mM (Figure 2).

### 2.2 Cell lines

The adenocarcinoma ovarian cancer cell line IGROV1 was cultured in Roswell Park Memorial Institute (RPMI) 1640 medium (Gibco, Life Technologies), malignant melanoma A-375 cell line (ATCC® CRL-1619™), hepatocellular carcinoma cell (HCC) lines Hep3B (ATCC® HB-8064™) and Huh7 (JCRB0403, JCRB Cell Bank) were cultured in Dulbecco's Modified Eagle's Medium (DMEM) (ATCC® 30–2002™), while HepG2 (ATCC® HB-8065™) and cervical adenocarcinoma cell line HeLa (ATCC® CCL-2™) were maintained in Eagle's Minimum Essential Medium (EMEM) (ATCC® 30–2003™). Culture of primary hepatocytes was provided in 96-well Clear Microplates by Karolinska Hospital (Stockholm, Sweden), preserved in William's E Medium (ThermoFisher, Catalogue #22551022) and treated within 24h. All media were supplemented with 10% FBS and cultures were incubated at 37 °C with 5% CO_2_.

### 2.3 Dose-response curve titration

The inhibitory concentration 50 (IC_50_) of selenofolate or folate was determined in serial dilution ranging (26, 39, 58, 88, 132, 197, 296, 444, 667, 1000, 1500 μM). A total number of 12,800 cells were seeded per well in 100 μL medium in Falcon® 96-well Clear Microplates (Corning®, catalogue #353072) 24h before treatment. Then, cells were treated with diluted compounds for 48h.

### 2.4 Cell viability test and IC_50_ determination

After 48h of treatment with selenofolate or folate, cell viability was measured using the Cell Titer-Glo® Luminescent Cell Viability Assay (Promega, Catalogue #G7570). Luminescence was read in a CLARIO star® (BMG LABTECH) multi-mode microplate reader. Viability levels were calculated in comparison to the untreated cells and subjected to dose-response curve fitting analyses in GraphPad Prism 8.3.0 Software (GraphPad Inc, USA) to calculate the IC_50_.

### 2.5 RNA extraction and RT-qPCR

RNA was isolated from cell pellets (1–3 × 10^6^ cells per pellet) using the RNeasy Plus Mini Kit (Qiagen). RNA concentrations were measured using a NanoDrop™ 1000 Spectrophotometer (ThermoFisher). Reverse transcription reactions of RNA (500–1000 ng) were performed using the Omniscript Reverse Transcription Kit (Qiagen) and oligo-dT prime in a final volume of 20 μl cDNA sample (1 μl) was subjected without further dilution to qPCR reactions. qPCR reactions were carried out in triplicates using the iQ SYBR® Green Supermix (BioRad) according to the manufacturer's instructions and measured in a CFX96 Touch Real-Time PCR Detection System (BioRad). Expression of the gene of interest was normalised to two housekeeping genes, primer sequences:

*ACTB*: F: 5′-AAAGACCTGTACGCCAACACA-3′, R: 5′-AGTACTTGCGCTCAGGAGGA-3’;

*HPRT*: F: 5′-GCAGACTTTGCTTTCCTTGG-3′, R: 5′-TATCCAACACTTCGTGGGGT-3’.

Primer sequences of genes of interest were designed using the NCBI Primer-BLAST online tool (www.ncbi.nlm.nih.gov/tools/primer-blast/):

*FOLR1*: F: 5′-CACAGCTGTCCCCTGGAATAAG-3′, R: 5′-TACTACAGCCACCCACACTAGAA-3’; *SLC19A1*: F: 5′-TACCTTTGCTTCTACGGCTTCAT-3′, R: 5′-GATCTCGTTCGTGACCTGCTC-3’; *SLC46A1*: F: 5′-TTAGTCATCACACCTGTCATCCG-3′, R: 5′-TGGGTAGAGTGAGTTGAAGATGC-3’;

### 2.6 siRNA transfection

Lipofectamine™ 3000 Transfection Reagent (ThermoFisher, Catalogue #L3000001) was used for siRNA transfection. Cells were seeded 24h before transfection in 6-well plates at a density that yielded approximate 70–80% confluency before transfection. 500 μl of transfection mix (5 μl Lipofectamine 3000 reagent and a final concentration of 15 nM siRNA in serum-free OptiMEM (Gibco, Life Technologies)) was incubated for 10 min at room temperature before adding to the cells, then incubated for 24h. Knockdown efficiency was assessed by RT-qPCR and cells were subjected to dose-response experiments in serial dilution ranging (26, 39, 58, 88, 132, 197, 296, 444, 667, 1000, 1500 μM) for 48h. Silencer Select® siRNAs (Ambion, Life Technologies) were reconstituted in sterile RNAse/DNAse-free H_2_O at a concentration of 5 μM *FOLR1* siRNAs: s5330, s5331, s5332; *SLC19A1* siRNA: s13085; *SLC46A1* siRNA: s41450; scrambled control siRNA: Silencer® was used as negative control.

### 2.7 Determination of apoptotic cell death

IGROV1 cells (10^6^ cells) were seeded in T25 flasks, then acclimatised for 24h before treatment. Subsequently, cells were treated with selenofolate (400 μM), approximate IC_50_ of selenofolate on IGROV1 cell line, for 24h and 48h. Apoptotic cell death was measured following the protocol of FITC Annexin V Apoptosis Detection Kit I (BD Pharmingen™, Catalogue # 556547) using BD FACSCanto™ II (BD Biosciences, USA). Untreated cells were used as negative control (NT control) and cells treated with staurosporine (2 μM) was used as a positive control.

### 2.8 Anti-proliferative assay

IGROV1 cells (50 × 10^3^) were seeded in 5 mL RPMI medium in T25 flasks. The cells were acclimatised for 24h before treatment. The cells then treated with selenofolate (400 μM) (approximate IC_50_ of selenofolate against IGROV1 cells). The number of cells was counted by the Trypan blue exclusion method at 24h, 48h, and 72h intervals adding equal parts of trypan blue solution (0.4 %) (Sigma, catalogue #T8154) to the cell suspension to obtain a 1: 2 dilution ratio before measuring in a TC20™ Automated Cell Counter (BioRAD). Untreated cells were used as negative control.

### 2.9 Statistical analysis

Data were analysed *vi*a one-way ANOVA and Nested t-test using GraphPad Prism 8.3.0 software (GraphPad Inc. USA) with α set to 0.05. Homogeneity of variance was ascertained using Brown–Forsythe test. The Tukey-Kramer HSD posthoc test was performed to determine the source of significance at *p ≤ 0.05*.

## 3 Results

### 3.1 FOLR1 expression in different cancer cell lines

**Figure 3:**
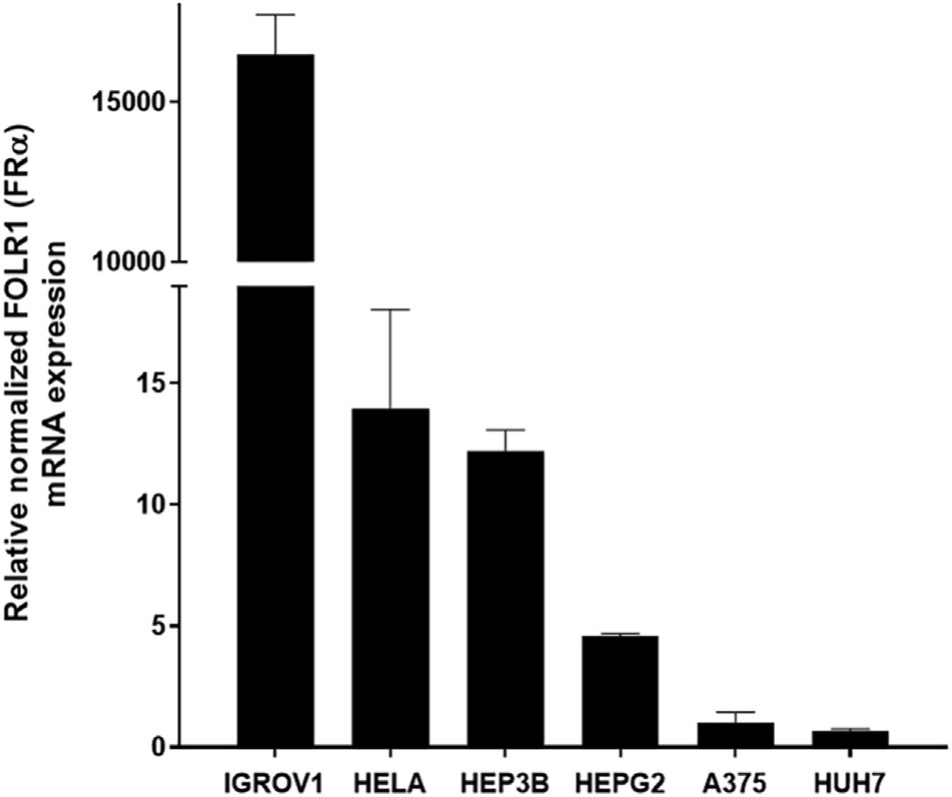
*FOLR1* expression in several cancer cell lines. Expression was normalised based on *ACTB* and *HPRT* housekeeping genes and demonstrated in comparison to the expression in the malignant melanoma cell line A375 (n = 3, mean ± SD).

To test the hypothesis whether selenofolate cytotoxicity is dependent on FRα expression, *FOLR1* mRNA expression status was measured by RT-qPCR, indicating that the IGROV1 cell line has the highest *FOLR1* expression, while HeLa cells and Hep3B cells expressed at an intermediate level, and the cell lines HepG2, Huh7 and A375 expressed *FOLR1* at low levels (Figure 3). We, therefore, focused our studies more on the IGROV1 cell line.

### 3.2 Selenofolate cytotoxicity in comparison to folate with differential FOLR1 expression

**Figure 4:**
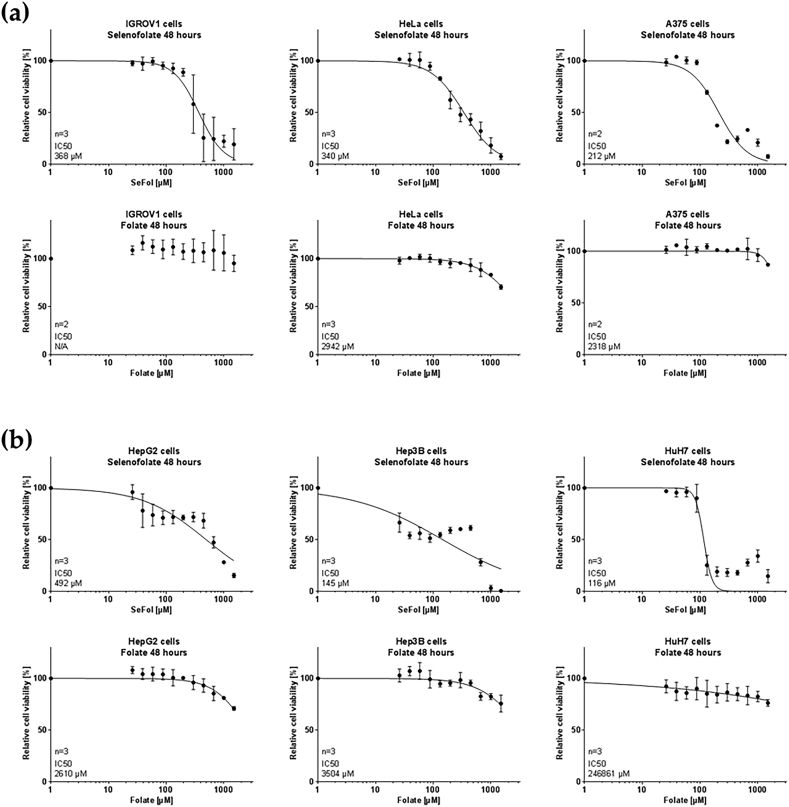
IC_50_ values of selenofolate (top panels) and folate (bottom panels) on (a) IGROV1, HeLa and A375 cell lines, and (b) HepG2, Hep3B and Huh7 cell lines (n = 3, mean ± SD).

The IC_50_ values of selenofolate in IGROV1, HeLa and A375 were similar (Figure 4). The calculated mean IC_50_ values were 368, 340, and 212 μM, respectively (Figure 4a), while folate alone in the same concentration range did not exert any cytotoxicity effect (Figure 4b). The calculated mean IC_50_ values for HepG2, Hep3B and Huh7 cell lines were 492, 145, and 116 μM, respectively (Figure 4b). Folate tested in the concentration range (0–1500 μM) also showed no cytotoxicity (Figure 4b).

### 3.3 Dose-response of selenofolate cytotoxicity against cells with siRNA mediated knockdown of transporter genes

**Figure 5:**
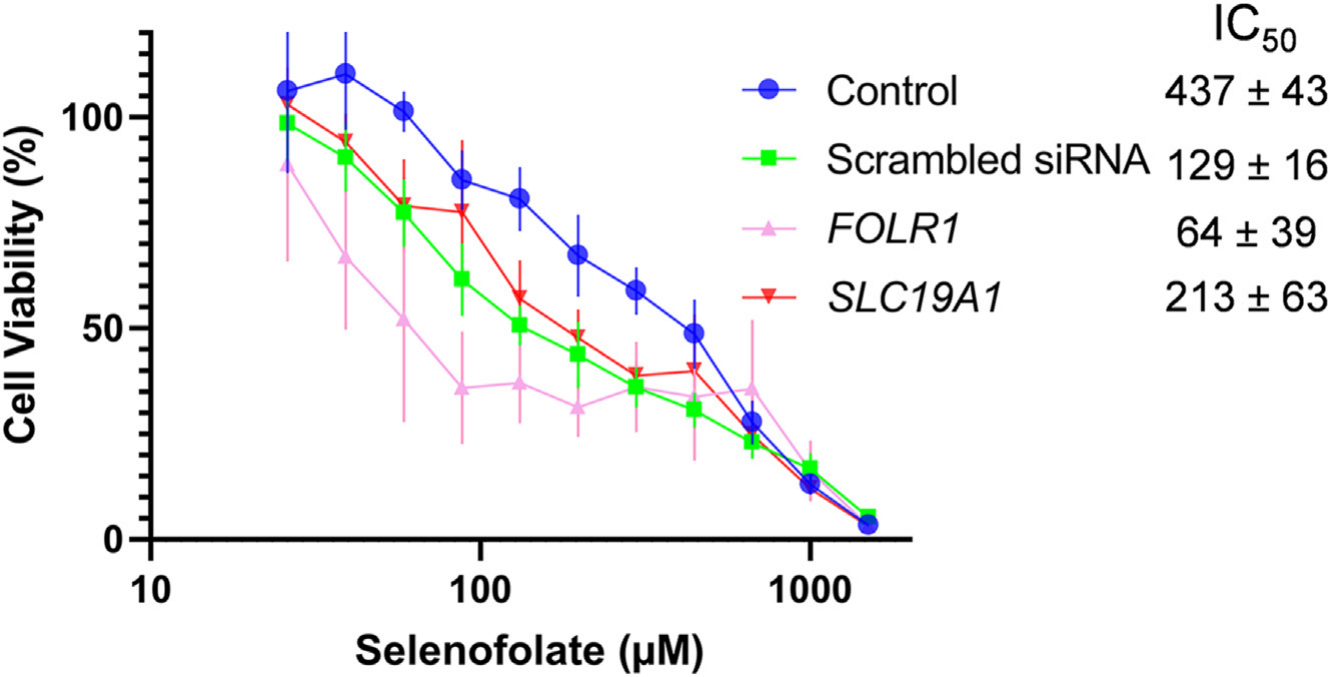
Dose-response curves and IC_50_ values (48h) of selenofolate on. IGROV1 cells after knockdown of siRNA *FOLR1* and *SLC19A1* (n = 6, Mean ± SD). Control; non-transfected cells. Scrambled siRNA; as negative control. The curves are an average of two separate experiments.

In IGROV1 cells, according to one-way ANOVA analysis, knockdown of *FOLR1* or *SLC19A1* did not affect the cytotoxic dose-response to selenofolate compared to scrambled siRNA as negative control. *i.e.* the IC_50_ of *FOLR1* or *SLC19A1* was statistically insignificant compared to negative control (*p* = 0.09) (Figure 5). This result indicates that neither *FOLR1* nor *SLC19A1* are involved in the transport of selenofolate into the IGROV1 cells.

### 3.4 Apoptotic effects of selenofolate

**Figure 6:**
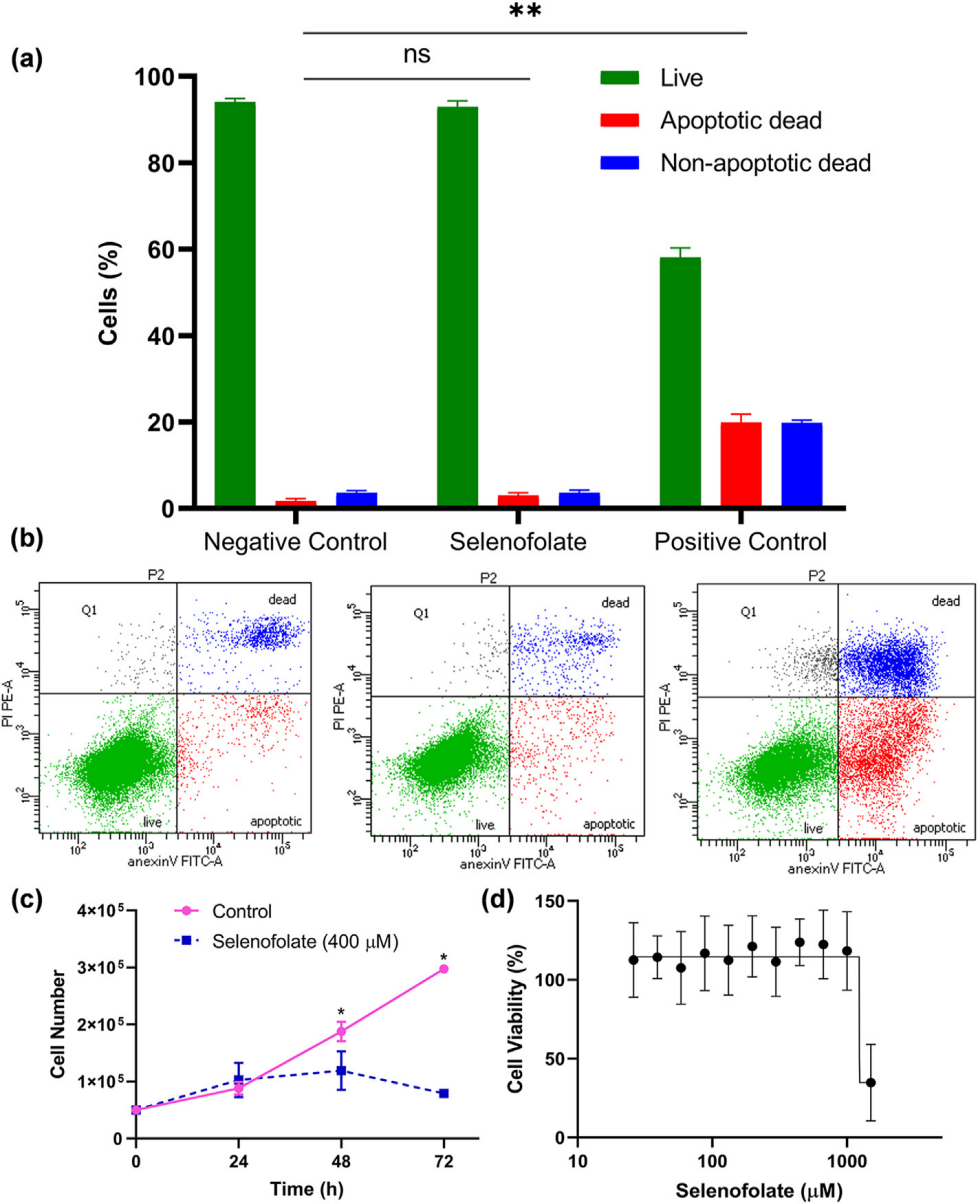
(a) Flow-cytometric percentage of live, apoptotic dead and non-apoptotic dead cells of IGROV1 cell line demonstrated as negative control (untreated cells), selenofolate treated (400 μM), and positive control (staurosporine treated (2 μM)) (n = 3, Mean ± SD). (b) Flow-cytometric quadrant graphs of IGROV1 cells after 48h, (left) negative control, (centre) selenofolate treated cells and (right) positive control. (c) Anti-proliferative effect of selenofolate on IGROV1 cell number measured in 24h, 48h and 72h intervals by Trypan blue exclusion method. Untreated cells were used as control (n = 3, Mean ± SD). (d) cytotoxicity of primary hepatocytes against a serial concentration of selenofolate (n = 8, Mean ± SD). The asterisk indicates significant difference *p ≤ 0.05* (∗), *p ≤ 0.01* (∗∗) or non-significant (ns).

Selenofolate treatment (400 μM), around the IC_50_ value for IGROV1 cells, did not show any statistical difference in apoptotic (~2%) and non-apoptotic cell death (~3%) in comparison to untreated IGROV1 cells (*p = 0.4*) using one-way ANOVA analysis (Figure 6a and b). Whereas staurosporine (2 μM), an apoptotic inducer (positive control), could increase both the apoptotic and non-apoptotic cell death by approximately 20% (Figure 6a and b) with statistical significance (*p < 0.01*). This result shows that the inhibitory effects of selenofolate are not applied through apoptotic cell death in the IGROV1 cell line.

### 3.5 Anti-proliferative effects of selenofolate

Using Nested t-test analysis showed a statistically significant difference in toxicity between non-treated control and selenofolate treated cells (*p* < 0.05). Selenofolate treatment showed an antiproliferative effect on the growth and cell number of IGROV1 cells after 72h in comparison to the non-treated control cells. The number of non-treated viable control cells was approximately 6-fold higher than the selenofolate treated cells after 72h indicating that selenofolate exerts an anti-proliferative effect on the growth and multiplication of IGROV1 cells (Figure 6c).

### 3.6 Effect of selenofolate on primary hepatocytes

A decrease in cell viability was observed in primary hepatocytes cells after 48h of treatment with a very high concentration of selenofolate (IC_50_ > 1500 μM) suggesting that toxicity of selenofolate is physiologically irrelevant on primary hepatocytes (Figure 6d).

## 4 Discussion

This study aimed to assess cancer cell toxicity *in vitro* using a covalent folate-conjugated selenium adduct (*i.e.* folate as a carrier for selenium) in light of the fact that rapidly dividing cancer cells often overexpress folate receptors and consume higher amounts of folate [5]. Folate targeting drugs are similar to the Trojan Horse story, a technique utilised for transporting anticancer agents into cancer cells [15]. Therapies targeting FRs are and have been developed, to include, Farletuzumab (Morphotek®, USA), Vintafolide and EC1456 (both from Endocyte Inc., USA) [16]. Noteworthy, previous research using Vintafolide; folate conjugated to the vinca alkaloid Desacetylvinblastine monohydrazide, led to regression of FR-expressing human tumour xenografts pre-clinically [17]. A Phase II clinical trial showed that patient survival benefited from Vintafolide treatment compared to historical controls [18]. Despite the early success of Vintafolide, the Phase III clinical trial of Vintafolide in combination with Doxil® was suspended in 2015 (NCT01170650) showing no life-extension benefit.

Our results show that treating cancer cells with selenofolate *in vitro* is an effective way to inhibit the growth of all cancer cells. Selenofolate did not exert apoptotic cell death in IGROV1 cells, whereas its antiproliferative effects are apparent (Figure 6). Upon analysis of these results, one might consider the following hypotheses. Selenium compounds forming selenides (RSe-) are known for their redox activity causing oxidative stress in cancer cells leading to cell death [2]. In pharmacological/higher doses, most selenium compounds (*e.g.,* selenite, selenocystine and methylseleninic acid) are known to increase metabolism producing metabolites (*e.g.,* monomethylselenol and hydrogen selenide) which are extremely redox reactive upon reaction with thiols exerting oxidative stress leading to cell death [2]. Previous studies using this selenofolate also demonstrated cytotoxic effects against triple-negative breast cancer cell line through the generation of oxidative stress [14]. However, since selenofolate did not induce cell death in IGROV1 cells, we conclude that the redox reaction of selenium leads to inhibition of cell growth in IGROV1 without apoptosis. For instance, it has been demonstrated that some selenium compounds like selenomethionine can cause cell cycle arrest in the G2-M phase of prostate cancer cells [19]. It would be of interest, therefore, to learn if the mechanism(s), in addition to oxidative stress, might be involved in the antiproliferative effect of selenofolate on IGROV1 cells.

Furthermore, the knockdown of expression of FRα and RFC1 did not change the sensitivity of IGROV1 cells to selenofolate (Figure 5). Therefore, the inhibitory effects of selenofolate on cancer cells could be independent of FRα endocytosis and its signalling/receptor pathway. The function of FRα, often overexpressed on cancer cells, is still poorly understood, but it has been proposed that transport of folate into the cancer cells is not a primary function of the FRα. For example, folic acid activates STAT3 through the FRα in a Janus Kinase-dependent pathway [9]. However, other folate transporters such as PCFT transporters might be involved in transporting selenofolate into these cells. Unlike high-affinity FRs that accumulate folates through endocytosis [20] folate transport systems, including RFC1 and PCFT have recently attracted attention for their ability to deliver antifolate drugs to cancer cells [21]. RFC1 and PCFT are functionally distinct in that RFC1 is optimal at neutral pH (~7.4) and is known as the main route of transport of most antifolates into tumour cells [20, 21, 22]. In contrast, PCFT functions optimally at acidic pH (~5.5–6.5) [20]. Selenofolate generating superoxide most likely accounts for its anti-proliferative activity in IGROV1 cells.

Finally, folate adducts such as 5-formyltetrahydrofolate (5-FTHF) (Leucovorin™), a Schiff-base adduct of tetrahydrofolate and formaldehyde [23], is used as a rescue therapy to decrease the adverse effects of medications such as methotrexate [24]. 5-FTHF is most efficiently transported into the cells through the PCFT receptor [25]. Thus, it is probable, that selenofolate, shown to redox cycle generating oxidative stress, is similarly transported through the low-affinity folate transporters such as PCFT.

## 5 Conclusion

The results of selenofolate treatment against a variety of cancer cells *in vitro* suggest that it may be used as a chemotherapeutic agent in human trials and thus provide a targeted selenium therapy to cancers overexpressing FRs with a high requirement for folate. FRα was not predominately involved in the transport of selenofolate into the IGRlOV1 cancer cells. Selenofolate did not exert its cytotoxic effects through apoptotic cell death under these experimental conditions in IGROV1 cells. Therefore, we suggest that the anti-proliferative activity is the main cause of the inhibitory effects of selenolofate on the growth of the IGROV1 cell line. However, future investigations will be required to investigate the mechanism of both the anti-proliferation effect and the cytotoxic safety of selenofolate as a viable pharmaceutical.

## Declarations

### Author contribution statement

Ali Razaghi; Antje Maria Zickler: Performed the experiments; Analyzed and interpreted the data; Wrote the paper.

Julian Spallholz: Conceived and designed the experiments; Wrote the paper.

Gilbert Kirsch: Contributed reagents, materials, analysis tools or data.

Mikael Björnstedt: Conceived and designed the experiments.

### Funding statement

This study was supported by 10.13039/501100002794Cancerfonden (180429), 10.13039/100016290Cancer- och Allergifonden (206), 10.13039/501100007232Radiumhemmets Forskningsfonder (171023) and 10.13039/501100004047Karolinska Institutet.

### Data availability statement

Data included in article/supplementary material/referenced in article.

### Declaration of interests statement

The authors declare the following conflict of interests: Mikael Björnstedt is listed as the inventor of a patent application concerning the use of intravenous inorganic selenium in the treatment of cancer patients. Mikael Björnstedt is also a shareholder in a company, SELEQ OY, which develops selenium formulations for supplements and pharmaceutical formulations for the treatment of cancer.

### Additional information

No additional information is available for this paper.
